# Blood types (ABO/Rhesus) and SARS-CoV-2 infection: a retrospective, cross-sectional study in 2828 healthcare workers

**DOI:** 10.2217/fvl-2022-0128

**Published:** 2022-10-17

**Authors:** Betul Copur, Serkan Surme, Ugurcan Sayili, Gulsah Tuncer, Melike Nur Ozcelik, Hulya Yilmaz-Ak, Muge Topal, Sumeyye Ustun-Al, Filiz Pehlivanoglu, Gonul Sengoz

**Affiliations:** ^1^Department of Infectious Diseases & Clinical Microbiology, Haseki Training & Research Hospital, Istanbul, 34096, Turkey; ^2^Department of Medical Microbiology, Institute of Graduate Studies, Istanbul University–Cerrahpasa, Istanbul, 34098, Turkey; ^3^Department of Public Health, Cerrahpasa Faculty of Medicine, Istanbul University–Cerrahpasa, Istanbul, 34098, Turkey; ^4^Department of Anesthesiology & Reanimation, Lutfi Kirdar Kartal Training & Research Hospital, Istanbul, 34865, Turkey; ^5^Infection Control Committee, Haseki Training & Research Hospital, Istanbul, 34096, Turkey

**Keywords:** ABO blood groups, healthcare workers, Rhesus status, SARS-CoV-2

## Abstract

**Background:** The authors aimed to investigate the relationship between ABO/Rhesus blood types and the risk of SARS-CoV-2 infection and hospitalization in healthcare workers (HCWs). **Materials & methods:** This study compared HCWs with (n = 510) and without (n = 2318) SARS-CoV-2 infection. Risk factors for SARS-CoV-2 infection and hospitalization in HCWs were shown as odds ratios with 95% CI. **Results:** Blood group O was found to be protective by 20% from the risk of developing SARS-CoV-2 infection in HCWs (29.2 vs 33.8%; odds ratio: 0.808; 95% CI: 0.655–0.996; p = 0.045). The prevalence of group O was lower in hospitalized patients than in outpatients (25 vs 29.5%; p = 0.614). **Conclusion:** These findings suggest that blood groups are associated with the development of SARS-CoV-2 infection.

## Plain language summary

In this study, the relationship between ABO blood groups and the development of SARS-CoV-2 infection was investigated in healthcare workers (HCWs) working in a tertiary hospital. A total of 2828 HCWs were included in the study. Of those, 510 (18%) had SARS-CoV-2 infection. The prevalence of blood group O was 33% (n = 933). The prevalence of group O was lower in hospitalized patients than in outpatients (25 vs 29.5%; p = 0.614). Further analysis revealed that blood group O was associated with a decreased risk of developing SARS-CoV-2 infection in HCWs by up to 20%.

## Tweetable abstract

In the study of Copur *et al.*, #bloodgroupO was found to be protective by 20% from the risk of developing #SARS-CoV-2 #infection in #healthcareworkers.

Identifying risk factors for COVID-19 caused by SARS-CoV-2 and severe disease is vital for the prevention of mortality and morbidity. Although age, comorbid diseases and smoking history are associated with the severity of COVID-19, it is thought that genetic factors may also be effective in the host thromboinflammatory response in patients with COVID-19 [1,2]. The relationship between viral infections and ABO blood groups has been known before [3,4]. For example, in the Middle East respiratory syndrome coronavirus epidemic, it was shown that those with the blood group O were less infected with the SARS coronavirus [5]. There are studies reporting a lower prevalence of COVID-19 in blood group O and a higher prevalence in blood group A. In addition, some studies suggest that severe/critical disease frequency is lower in blood type O as well [6–10]. Moreover, some studies found a relationship between the presence of anti-A and mild COVID-19 [11,12]. Another study reported that the differences between cytokine levels in some blood types may be critical for poor outcomes such as the need for the intensive care unit (ICU) and mechanical ventilation support or death due to COVID-19 [13].

Although the above-mentioned studies have reported that ABO/Rhesus (Rh) blood groups are associated with the risk of SARS-CoV-2 infection and disease severity, a limited number of studies show the relationship between SARS-CoV-2 infection and blood groups in healthcare workers (HCWs). In this study, the authors aimed to investigate the relationship between ABO/Rh blood types and the risk of SARS-CoV-2 infection and hospitalization in HCWs in a tertiary hospital.

## Materials & methods

### Study design & participants

**Figure 1.**
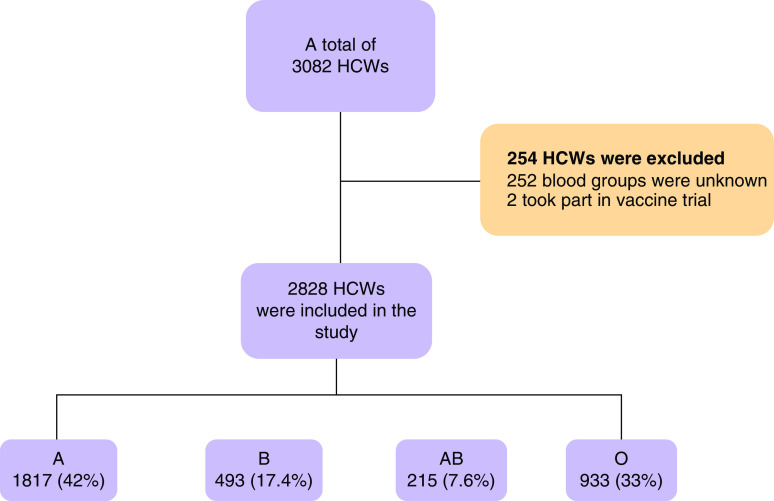
The flowchart showing the included and excluded HCWs and ABO blood groups distrubitions. HCW: Healthcare worker.

**Figure 2.**
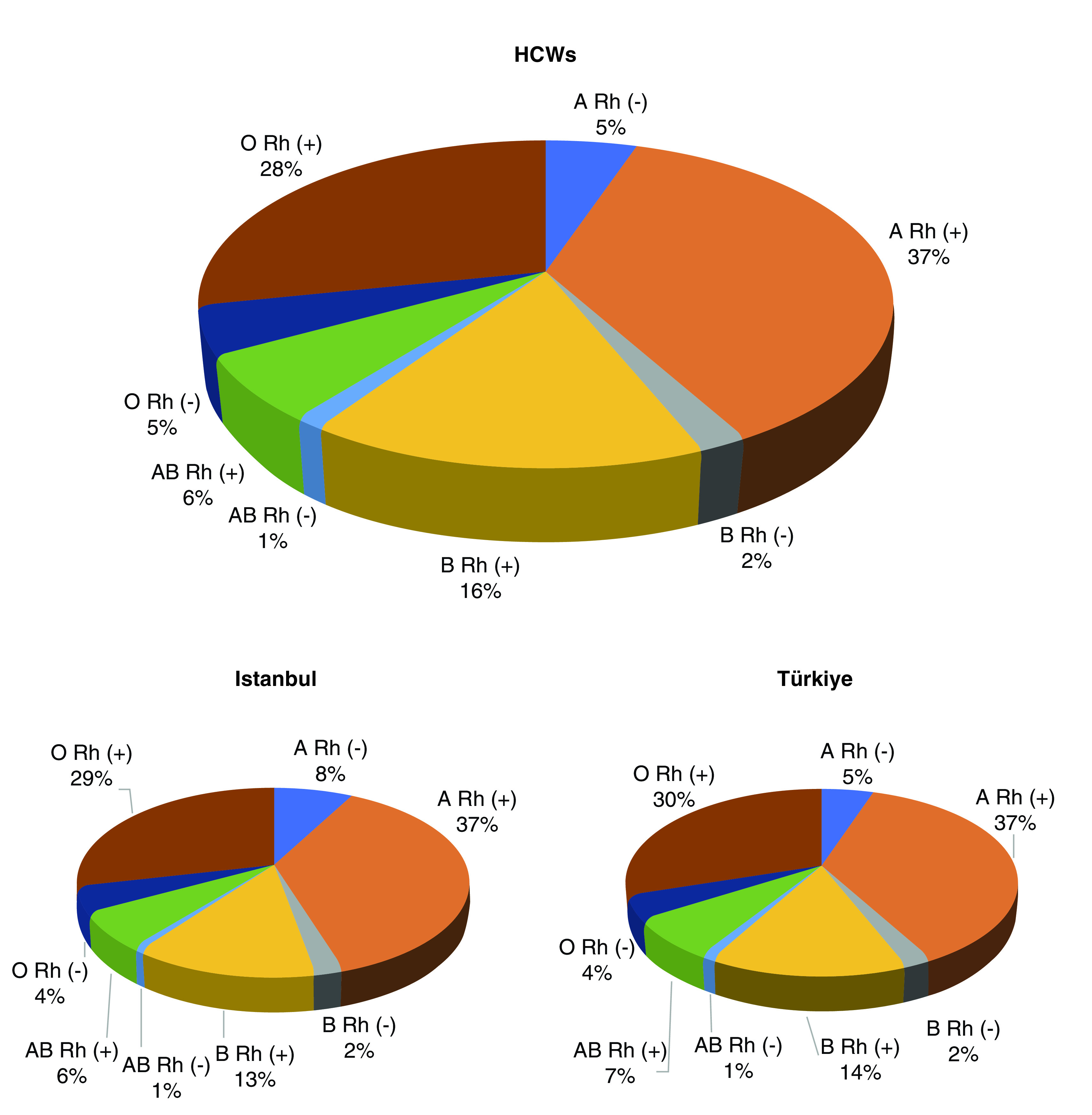
ABO blood groups distributions of HCWs and the general population. HCW: Healthcare worker.

This single-center, retrospective study was carried out between 11 March 2020 and 15 January 2021 at Haseki Training and Research Hospital (Istanbul, Turkey) when there was no vaccination program for COVID-19 in the country yet. The authors' 800-bed-capacity tertiary care hospital was designed as a pandemic epicenter during the COVID-19 crisis. A total of 3082 HCWs who were employed at Haseki Training and Research Hospital were evaluated. Two-hundred fifty-two HCWs were excluded due to missing ABO/Rh blood group data. In addition, two HCWs who participated in the vaccine trial were excluded. Finally, 2828 HCWs met the inclusion criteria (Figure 1). The blood group distributions of the study group comprising HCWs in the hospital were comparable with those of the individuals in the province and country (Figure 2) [14,15].

Demographic characteristics, blood groups, smoking status, chronic diseases and SARS-CoV-2 PCR results of the HCWs participating in the study were obtained retrospectively from the hospital system. HCWs with SARS-CoV-2 infection (n = 510) were compared with those who were not infected (n = 2318). To determine the risk factors for hospitalization, HCWs with SARS-CoV-2 infection were divided into groups as hospitalized (n = 28) and nonhospitalized (n = 482), and the groups were compared using statistical methods. All patients with pneumonia were hospitalized and followed up in the hospital.

### Definition of SARS-CoV-2 infection

Patients with symptoms in whom a SARS-CoV-2 PCR was positive in respiratory samples and/or patients with COVID-19-specific radiological findings were classified as having SARS-CoV-2 infection. Negative samples were repeated after 48–72 h. According to the guidelines issued by the General Directorate of Public Health of the Turkish Ministry of Health for COVID-19, patients with at least one of the signs and symptoms of fever or acute cough and respiratory distress, a history of traveling abroad in the past 14 days or of close contact with a confirmed COVID-19 case or the presence of hospitalization for severe acute respiratory infections and the absence of an explanation for the clinical manifestation with another cause/disease were defined as probable cases. Patients diagnosed with SARS-CoV-2 infection with respiratory distress (respiratory rate per minute <24, saturation >93%) and no pulmonary involvement were considered to have uncomplicated SARS-CoV-2 infection. Patients with fever, muscle/joint pain, cough, sore throat and nasal congestion, tachypnea (≥30/min), SpO2 less than 90% on room air, poor prognostic values in blood work on admission (lymphocyte count <800/μl, C-reactive protein >40 mg/l, ferritin >500 ng/ml, d-dimer >1000 ng/ml etc.) and bilateral diffuse pneumonia on chest radiography or CT scan were defined as having severe pneumonia. Lung involvement without evidence of severe pneumonia was classified as mild-to-moderate pneumonia [16].

### Diagnostic methods

Samples were obtained with synthetic fiber swabs and placed in a sterile transfer tube containing a viral nucleic acid transport medium (Biospeedy, Bioexen, Istanbul, Turkey). Samples were stored at 2–8°C until they were transferred to the microbiology laboratory and processed. Since the maximum time for SARS-CoV-2 RNA extraction in transport tubes is 5 min, the PCR step was performed without intermediate processing. The Biospeedy SARS-CoV-2 Double Gene RT-qPCR, version 4 (Biospeedy, Bioexen, Istanbul, Turkey) kit was used with a step reverse transcription-PCR targeting the SARS-CoV-2-specific N gene and the *ORF1AB* gene region. The oligo mix contained an extraction control and an internal control targeting the human RNase P gene. Negative and positive controls were used for DNA isolation. A cycle threshold value of less than 40 was considered to be positive.

### Statistical analysis

IBM SPSS Statistics for Windows was used for statistics. Categorical variables were expressed as frequencies (n) and percentages (%), while numerical variables were expressed as medians (interquartile range). The Mann–Whitney U test, since normal distribution conditions were not met in the analysis of continuous variables; χ^2^ test; or Fisher's exact test was used in the analysis of categorical variables. The relative risk was shown as the odds ratio (OR). p < 0.05 was considered statistically significant.

## Results

A total of 2828 HCWs were included in the study. Of those, 59.3% (n = 1678) were female, 40.7% (n = 1150) were male and the median age was 30 years (26–40). A total of 1187 (42%) patients had blood group A, 493 (17.4%) had blood group B, 215 (7.6%) had blood group AB, 933 (33%) had blood group O and 2464 (87.1%) were Rh-positive (Table 1). The distribution of HCWs was as follows: 650 (23%) doctors, 1130 (40%) nurses/medical technicians, 368 (13%) security/medical secretaries, 622 (22%) cleaning personnel and 58 (2%) others (Table 2). Among HCWs, 510 (18%) people were infected with SARS-CoV-2, while 28 (1%) were hospitalized. Age (p = 0.627) and sex (p = 0.098) characteristics were similar in the groups with and without SARS-CoV-2 infection. Of 1416 HCWs, 877 (61.9%) had never smoked. The incidence of SARS-CoV-2 infection was similar among nonsmokers, active smokers and ex-smokers (p = 0.561). The frequency of chronic disease in HCWs who had SARS-CoV-2 infection was significantly lower than that in HCWs who were not infected with SARS-CoV-2 (11.4% vs 27.4%; p < 0.001). The prevalence of chronic diseases such as hypertension (7.1% vs 15.8%; p < 0.001), coronary artery disease (0.8% vs 4.9%; p < 0.001) and diabetes mellitus (2.7% vs 10.0%; p < 0.001) was lower in those with SARS-CoV-2. The proportions of HCWs carrying Rh (p = 0.731), A (p = 0.278), B (p = 0.690) and AB (p = 0.335) groups were similar in groups with and without SARS-CoV-2 infection. Although the risk of SARS-CoV-2 infection was lower in HCWs who had anti-A compared with those who did not, this difference was not statistically significant (48.6% vs 51.1%; p = 0.308; Table 3). When blood groups were examined, the rate of those with O blood type was found to be significantly lower in those who had SARS-CoV-2 infection than in those who did not have O blood type (15.6% vs 23.5%; OR: 0.808; 95% CI: 0.655–0.996; p = 0.045; Tables 3 & 4). The prevalence of blood group A (50% vs 43.8%; p = 0.519) was higher in hospitalized patients than in outpatients. The prevalence of group O was lower in hospitalized patients than in outpatients (25% vs 29.5%; p = 0.614). However, these differences did not reach statistical significance (Table 5). Of 28 hospitalized patients, eight had severe pneumonia and 20 had mild-to-moderate pneumonia. Severe pneumonia developed in three patients from blood group A (0.25%), two patients from group B (0.45%), two patients from group AB (0.93) and one patient from group O (0.10). Due to SARS-CoV-2 infection, three patients were followed up in the ICU with invasive mechanical ventilation support. Two of the three HCWs admitted to the ICU died. Two of the three patients who needed mechanical ventilation were blood group A. Two deceased patients had group A as well (Table 6).

**Table 1.** Demographic characteristics of healthcare workers with ABO blood groups.

	Total n (%)	A n (%)	B n (%)	AB n (%)	O n (%)	p-value
	2828 (100)	1187 (42)	493 (17.4)	215 (7.6)	933 (33)	
Age, median (interquartile range)	30 (26–40)	31 (26–41)	30 (26–38.5)	32 (27–40)	31 (26–39)	0.068^§^
Gender						
Male, n (%)	1150 (40.7)	495 (41.7)	187 (37.9)	81 (37.7)	387 (41.5)	0.375^†^
Female, n (%)	1678 (59.3)	692 (58.3)	306 (62.1)	134 (62.3)	546 (58.5)	
Smoking status	1416					
Active smoker, n (%)	511 (36.1)	224 (36.7)	82 (34.5)	41 (36.6)	164 (36.0)	0.458^‡^
Never used, n (%)	877 (61.9)	371 (60.8)	149 (62.6)	69 (61.6)	288 (63.2)	
Ex-smoker, n (%)	28 (2.0)	15 (2.5)	7 (2.9)	2 (1.8)	4 (0.9)	
Chronic disease, n (%)	692 (24.5)	301 (25.4)	111 (22.5)	62 (28.8)	218 (23.4)	0.225^†^
Diabetes mellitus, n (%)	246 (8.7)	105 (8.8)	34 (6.9)	30 (14.0)	77 (8.3)	0.021^†^
Hypertension, n (%)	402 (14.2)	171 (14.4)	73 (14.8)	34 (15.8)	124 (13.3)	0.733^†^
Congestive heart failure, n (%)	15 (0.5)	4 (0.3)	2 (0.4)	1 (0.5)	8 (0.9)	0413^‡^
Cerebrovascular disease, n (%)	6 (0.2)	2 (0.2)	2 (0.4)	1 (0.5)	1 (0.1)	0.334^‡^
Chronic obstructive pulmonary disease, n (%)	32 (1.1)	12 (1.0)	7 (1.4)	3 (1.4)	10 (1.1)	0.803^‡^
Chronic hepatitis B, n (%)	71 (2.5)	29 (2.4)	9 (1.8)	3 (1.4)	30 (3.2)	0.267^†^
Coronary artery disease, n (%)	118 (4.2)	46 (3.9)	22 (4.5)	12 (5.6)	38 (4.1)	0.692^†^
Peripheral artery disease, n (%)	8 (0.3)	3 (0.3)	1 (0.2)	0 (0)	4 (0.4)	0.857^‡^
Rhesus status						
Rh+, n (%)	2464 (87.1)	1044 (88)	439 (89)	181 (84.2)	800 (85.7)	0.133^†^
Rh-, n (%)	364 (12.9)	143 (12)	54 (11)	34 (15.8)	133 (14.3)	

† χ^2^ test.

‡ Fisher's exact test.

§ Kruskal–Wallis test.

**Table 2.** Distribution of the occupations of healthcare workers.

Job	n = (%)
Doctor	650 (22.9)
Nurse/medical technician	1130 (39.9)
Security/medical secretary	368 (13.0)
Cleaning personnel	622 (21.9)
Others	58 (2.0)

**Table 3.** Association between ABO/Rhesus blood groups and SARS-CoV-2 infection in healthcare workers.

	SARS-CoV-2 infection (+) n = 510 (%)	SARS-CoV-2 infection (-) n = 2318 (%)	χ^2^ coefficient	p-value	Odds ratio (95% CI)
Age, median (interquartile range)	30 (26–39)	31 (26–40)		0.627^§^	
Gender					
Male, n (%)	224 (43.9)	926 (39.9)	2.735	0.098^†^	1.177 (0.97–1.429)
Female, n (%)	286 (56.1)	1392 (60.1)			
Rhesus status					
Rh+, n (%)	442 (86.7)	2022 (87.2)	0.118	0.731^†^	0.952 (0.717–1.263)
Rh-, n (%)	68 (13.3)	296 (12.8)			
Group A and others					
A, n (%)	225 (44.1)	962 (41.5)	1.175	0.278^†^	1.113 (0.917–1.350)
Non-A, n (%)	285 (55.9)	1356 (58.5)			
Group B and others					
B, n (%)	92 (18)	401 (17.3)	0.159	0.690^†^	1.052 (0.819–1.351)
Non-B, n (%)	418 (82)	1917 (82.7)			
Group AB and others					
AB, n (%)	44 (8.6)	171 (7.4)	0.930	0.335^†^	1.186 (0.839–1.676)
Non-AB, n (%)	466 (91.4)	2147 (92.6)			
Group O and others					
O, n (%)	149 (29.2)	784 (33.8)	4.013	**0.045** ^†^	**0.808 (0.655–0.996)**
Non-O, n (%)	361 (70.8)	1534 (66.2)			
Anti-A (groups B and O)					
Anti-A (+), n (%)	248 (48.6)	1185 (51.1)	1.040	0.308^†^	0.905 (0.747–1.096)
Anti-A (-), n (%)	262 (51.4)	1133 (48.9)			
Anti-B (groups A and O)					
Anti-B (+), n (%)	376 (73.7)	1746 (75.3)	0.570	0.450^†^	0.919 (0.739–1.144)
Anti-B (-), n (%)	134 (26.3)	572 (24.7)			
Smoking status					
Active smoker, n (%)	89 (34.4)	422 (36.5)	1.156	0.561†	0.924 (0.695–1.228)
Never used, n (%)	163 (62.9)	714 (61.7)			reference
Ex-smoker, n (%)	7 (2.7)	21 (1.8)			1.460 (0.610–3.493)
Chronic disease, n (%)	58 (11.4)	634 (27.4)	57.74	**<0.001** ^†^	**0.341 (0.255–0.455)**
Diabetes mellitus, n (%)	14 (2.7)	232 (10.0)	27.769	**<0.001** ^†^	**0.254 (0.147–0.439)**
Hypertension, n (%)	36 (7.1)	366 (15.8)	26.130	**<0.001** ^†^	**0.405 (0.284–0.579)**
Congestive heart failure, n (%)	3 (0.6)	12 (0.5)	0.039	0.742^‡^	1.137 (0.320–4.044)
Cerebrovascular disease, n (%)	0 (0)	6 (0.3)	1.323	0,599^‡^	–
Chronic obstructive pulmonary disease, n (%)	6 (1.2)	26 (1.1)	0.011	0.916^†^	1.049 (0.430–2.563)
Chronic hepatitis B, n (%)	8 (1.6)	63 (2.7)	2.256	0.133^†^	0.570 (0.272–1.198)
Coronary artery disease, n (%)	4 (0.8)	114 (4.9)	17.865	**<0.001**	**0.153 (0.056–0.416)**
Peripheral artery disease, n (%)	0 (0)	8 (0.3)	1.765	0.365^‡^	–

† χ^2^ test.

‡ Fisher's exact test.

§ Mann–Whitney U test.

The p-values less than 0.05 in bold are statistically significant.

**Table 4.** Relationship with SARS-CoV-2 infection in O and non-O blood groups.

	O n = 933 (%)	Non-O n = 1895 (%)	χ^2^ coefficient	p-value	Odds ratio (95% CI)
SARS-CoV-2 infection (+)	149 (15.6)	361 (23.5)	4.013	**0.045**	**0.808 (0.655–0.996)**
SARS-CoV-2 infection (–)	784 (84.4)	1534 (73.5)			

The p-values less than 0.05 in bold are statistically significant.

**Table 5.** Association between ABO/Rhesus blood groups and SARS-CoV-2 infection requiring hospitalization in healthcare workers.

	SARS-CoV-2 infection (+)		χ^2^ coefficient	p-value	Odds ratio (95% CI)
	Hospitalization n = 28 (%)	Without hospitalization n = 482 (%)			
Age, median (interquartile range)	36 (26.5–43.8)	30 (26–39)		0.087^§^	
Gender					
Male, n (%)	12 (42.9)	212 (44.0)	0.014	0.907^†^	0.955 (0.442–2.063)
Female, n (%)	16 (57.1)	270 (56.0)			
Rhesus status					
Rh+, n (%)	26 (92.9)	416 (86.3)	0.983	0.564^‡^	2.062 (0.478–8.894)
Rh-, n (%)	2 (7.1)	66 (13.7)			
Group A and others					
A, n (%)	14 (50.0)	211 (43.8)	0.416	0.519^†^	1.284 (0.599–2.753)
Non-A, n (%)	14 (50.0)	271 (56.2)			
Group B and others					
B, n (%)	2 (7.1)	90 (18.7)	2.1379	0.123^†^	0.335 (0.078–1.437)
Non-B, n (%)	26 (92.9)	392 (81.3)			
Group AB and others					
AB, n (%)	5 (17.9)	39 (8.1)	3.202	0.083^‡^	2.469 (0.890–6.855)
Non-AB, n (%)	23 (82.1)	443 (91.9)			
Group O and others					
O, n (%)	7 (25.0)	142 (29.5)	0.255	0.614^†^	0.798 (0.332–1.919)
Non-O, n (%)	21 (75.0)	340 (70.5)			
Anti-A (groups B and O)					
Anti-A (+), n (%)	9 (32.1)	239 (49.6)	3.223	0.073^†^	0.482 (0.214–1.086)
Anti-A (-), n (%)	19 (67.9)	243 (50.4)			
Anti-B (groups A and O)					
Anti-B (+), n (%)	21 (75.0)	355 (73.7)	0.025	0.875^†^	1.073 (0.446–2.585)
Anti-B (-), n (%)	7 (25.0)	127 (26.3)			
Smoking status					
Active smoker, n (%)	4 (22.2)	85 (35.3)	2.030	0.518^‡^	0.501 (0.160–1.570)
Never used, n (%)	14 (77.8)	149 (61.8)			reference
Ex-smoker, n (%)	0 (0)	7 (2.9)			–
Chronic disease, n (%)	4 (14.3)	54 (11.2)	0.249	0.545^‡^	1.321 (0.442–3.951)
Diabetes mellitus, n (%)	2 (7.1)	12 (2.5)	2.146	0.176^‡^	3.013 (0.641–14.169)
Hypertension, n (%)	2 (7.1)	34 (7.1)	0	1^‡^	1.014 (0.231–4.452)
Congestive heart failure, n (%)	0 (0)	3 (0.6)	0.175	1^‡^	–
Cerebrovascular disease, n (%)	0 (0)	0 (0)	NA	NA	–
Chronic obstructive pulmonary disease, n (%)	0 (0)	6 (1.2)	0.353	1^‡^	–
Chronic hepatitis B, n (%)	0 (0)	8 (1.7)	0.472	1^‡^	–
Coronary artery disease, n (%)	0 (0)	4 (0.8)	0.234	1^‡^	–
Peripheral artery disease, n (%)	0 (0)	0 (0)	NA	NA	–

† χ^2^ test.

‡ Fisher's exact test.

§ Mann–Whitney U test.

NA: Not applicable.

The p-values less than 0.05 in bold are statistically significant.

**Table 6.** ABO blood groups and prognosis in SARS-CoV-2 infection.

	A n = 1187 (42%)	B n = 439 (17.4%)	AB n = 215 (7.6%)	O n = 933 (33%)
SARS-CoV-2 infection	225 (18.95)	92 (20.95)	44 (20.46)	149 (15.96)
Hospitalization	14 (1.17)	2 (0.45)	5 (2.32)	7 (0.75)
Length of hospital stay (days)	6.6 ± 4.1	9 ± 5.5	6.1 ± 2.7	5.9 ± 2.6
Severe pneumonia	3 (0.25)	2 (0.45)	2 (0.93)	1 (0.10)
Mechanical ventilation	2 (0.16)	0 (0.00)	1 (0.46)	(0.00)
Need for intensive care unit	2 (0.16)	0 (0.00)	1 (0.46)	(0.00)
Recovery	223 (18.78)	92 (20.95)	44 (20.46)	149 (15.96)
Death	2 (0.16)	0 (0.00)	0 (0.00)	(0.00)

## Discussion

In this study, the relationship between blood groups (ABO/Rh) and SARS-CoV-2 infection was investigated among HCWs working in a tertiary hospital, and it was observed that SARS-CoV-2 infection developed at an ~20% lower rate in blood group O (15.6% vs 23.5%; OR: 0.808; 95% CI: 0.655–0.996; p = 0.045). In addition, the prevalence of group O was found to be lower in patients hospitalized due to SARS-CoV-2 infection than in nonhospitalized patients (25% vs 29.5%). Most likely, due to the low number of hospitalized patients, no significant difference was found (p = 0.614).

The distributions of ABO blood groups in HCWs were similar to those of individuals in the province and country [14,15]. Of the blood donors of the Turkish Red Crescent Blood Center in 2005–2012, 38% had blood group A, 34% had blood group O, 16% had blood group B and 8% had blood group AB [14]. In another study conducted in Istanbul, the blood groups of 123,900 people were examined and it was reported that 33.8% of the people had blood group O, 42.8% had blood group A, 15.3% had blood group B and 7.1% had blood group AB [15].

The majority of the studies on ABO/Rh blood groups have included data from transfusion records. However, the present study enrolled HCWs who were closely followed-up for COVID-19. When examining the literature, despite different results, it was generally found that the risk of SARS-CoV-2 infection was high in blood group A, while blood group O was protective against SARS-CoV-2 infection [8,9]. While Zhao *et al.* found the risk of SARS-CoV infection to be 1.3-fold higher in blood group A, they found blood group O to be 32% protective against SARS-CoV-2 infection [8]. In a similar study conducted in Turkey, the prevalence of blood group A was higher in patients with SARS-CoV-2 infection than in the control group, whereas the prevalence of blood group O was lower. In the same study, blood groups were not associated with the prognosis of COVID-19 [9]. In a Canadian study, O blood and Rh- were associated with the reduced development of SARS-CoV-2 infection and severe COVID-19 [10]. In another study, blood type A was associated with increased SARS-CoV-2 infection, while blood type O was not associated with SARS-CoV-2 infection [17]. Like the results of the present study, Latz *et al.* did not find blood type A to be associated with infection risk (OR: 1.00; 95% CI: 0.88–1.13) but showed a reduced risk of COVID-19 in blood group O (OR: 0.84; 95% CI: 0.75–0.95). The same study reported that Rh status, B and AB groups were associated with increased COVID-19 [18]. According to the results of the present study, the risk of SARS-CoV-2 infection was lower in blood group O (15.6% and 23.5%; OR: 0.808; 95% CI: 0.655–0.996; p = 0.045); Rh status and other blood groups (A, B, AB) were not found to be associated with the risk of SARS-CoV-2 infection.

Anti-A and anti-B antibodies have been shown to be one of the causes of SARS-CoV-2 infection susceptibility, which varies according to blood groups. One of the hypotheses on the subject is that anti-A antibodies inhibit the binding of SARS-CoV-2 spike protein and ACE-2 receptor, preventing the adhesion of the virus to the cell and thus causing viral neutralization [11]. Gérard *et al.* examined the Zhao *et al.* study, which has the largest patient population in the literature, from a different perspective and investigated the relationship between the presence of anti-A and COVID-19. They compared 1888 COVID-19 patients with 3694 controls. The researchers concluded that anti-A presence was lower in COVID-19 patients than in patients without COVID-19, while there was no difference in anti-B [12]. In a study comparing 430 patients with COVID-19 and 2212 healthy blood donors, male sex and advanced age were found to be risk factors for SARS-CoV-2 infection, while the anti-A group was found to be protective against SARS-CoV-2 infection (OR: 0.62; 95% CI: 0.50–0.78; p < 0.001) [19]. In the present study, the anti-A (B/O) group (48% vs 51%; p = 0.308) and anti-B (O/B) group (73% vs 75%; p = 0.450) did not increase the risk for the development of SARS-CoV-2 infection. Similarly, Almadhi *et al.* showed that there was no relationship between A and B group antibodies and infection [20].

Although a high ACE-2 receptor level increases SARS-2 susceptibility [21], there are reports showing that it may be protective against cardiovascular disease and severe SARS-CoV-2 [22]. The protective effect of blood group O on severe SARS-CoV-2 infection may be due to the presence of lower ACE and higher ACE-2 levels in blood group O due to the absence of some ABO polymorphism genes. It has been reported that more IL-6 is released in blood group O and that this cytokine causes an increase in ACE-2 levels by inhibiting ACE [23]. The A antigen in the A blood group creates endothelial damage as a result of increased ACE system activation and adhesion molecule release (P-selectin, ICAM-1), causing a susceptibility to thromboinflammatory events and an increased risk of serious SARS-CoV-2 infection [24,25].

There are different reports on the relationship between the prognosis of COVID-19 and blood groups (ABO/Rh). Wu *et al.* stated that there is no relationship between blood types and severe COVID-19 [26]. Muñiz-Diaz and colleagues reported that mortality was higher in the A blood group than in the O blood group and that the O and A blood groups were two important blood groups in determining the prognosis of the patients with COVID-19 [27]. Serum inflammatory markers C-reactive protein, procalcitonin, d-dimer, lymphocyte count, ferritin and albumin are closely related to the severity of SARS-CoV-2 infection [28–33]. Another hypothesis states that the relationship between blood groups and serum levels of inflammatory cytokines may determine disease severity [13,28]. In one study, the risk of mechanical ventilation or death was found to be more than twofold lower in patients with blood type O, and it was shown that only patients with blood type O were able to generate a good immune response with effective cytokine release. In this study, the levels of 45 different cytokines were measured in the serum of 108 patients with COVID-19 at the first admission and on the sixth day thereafter. It was found that all cytokine levels, except for HGF, were significantly higher in patients with blood group O than in the others. The authors concluded that higher cytokine levels were more clinically associated with blood group O than others [13]. In another study conducted with COVID-19 patients monitored in the ICU, it was pointed out that the need was greater for more mechanical ventilation (p = 0.02) and continuous renal replacement therapy (p = 0.04) and the ICU length of stay was longer (p = 0.03) in groups B and AB. However, when the researchers compared groups O and B with groups A and AB, they showed that there was no difference in the serum levels of inflammatory cytokines [28]. According to the present study's results, the hospitalization rate was higher in patients with COVID-19 with blood groups A and AB, whereas this rate was lower in blood groups O and B. However, the authors could not statistically prove the relationship between blood types and hospitalization, which may be due to the small number of hospitalized patients with COVID-19. Statistical analysis of mortality could not be performed because only three subjects were admitted to the ICU during the study of HCWs in the hospital. Two of the three subjects who required mechanical ventilation and were admitted to the ICU had blood type A (67%).

SARS-CoV-2 infection risk factors have been investigated since the early stages of the pandemic, and sex, age, and comorbid conditions have been reported as host factors in SARS-CoV-2 infection and prognosis [2,34–36]. Advanced age may contribute to the severity of SARS-CoV-2 infection by creating negative effects, particularly on the target organ, the lung [33]. In a meta-analysis, age greater than 70 years and male sex were found to be associated with a higher risk of SARS-CoV-2 infection [34]. Among the reasons for the lower risk of disease in women is that they are more likely to maintain social distance and hygiene. In addition, it can be hypothesized that the estradiol hormone in women increases the amount of ADAM17 and thus ACE-2, which prevents SARS-CoV-2 from entering the host cell [36]. According to the results of the present study, sex and age did not cause a difference in the development of SARS-CoV-2 infection and hospitalization rates. This may be because the HCWs participating in the study were not advanced in age (median age was 30 years [26–40]). It is known that active smoking increases susceptibility to lung infections. However, the relationship of smoking to the risk of COVID-19 pneumonia and serious illness is not clear [37–40]. In a study conducted in Turkey country with outpatients who had mild-to-moderate COVID-19, it was reported that pneumonia occurred more frequently in nonsmokers [38]. In one meta-analysis, smoking was associated with a 1.9-fold increased risk for the progression of COVID-19 [39]. Another meta-analysis showed that active smoking was not associated with the prognosis of COVID-19 disease. Four of the five studies included in the analysis found no association between severe COVID-19 and active smoking [40]. Being a nonsmoker in the hospital in the present study was not found to be protective against the development of SARS-CoV-2 infection (62.9% vs 61.7%; p = 0.561) or related hospitalization (77.8% vs 61.8%; p = 0.875).

The prevalence of comorbidities, especially hypertension and diabetes mellitus, is high in those with SARS-CoV-2 infection [41–45]. In a study examining 5700 patients hospitalized for SARS-CoV-2 infection, 89.3% had at least one chronic condition, with hypertension, obesity and diabetes mellitus ranking in the top three [44]. The frequency of coronary artery disease has been shown to be lower than that of diabetes mellitus and hypertension, but its contribution to mortality is higher [44,45]. In the present study, contrary to the literature data, the frequency of hypertension, coronary artery disease and diabetes mellitus, which are comorbid diseases, was found to be lower in those who had SARS-CoV-2 infection, while the frequency of chronic diseases was found to be similar between those with COVID-19 requiring hospitalization and outpatients (Tables 3 & 5). This can be explained by the fact that HCWs who had a chronic disease in the first months of the pandemic better complied with social distancing and hygiene rules. In addition, HCWs with comorbidities were kept away from risky practices and were less in contact with social life.

This study had several strengths. First, the HCWs included in the study had a blood group distribution similar to that of the population of the province and country. Second, the authors strictly followed the recommendations of the Turkish Ministry of Health COVID-19 Guidelines for HCWs with close contact with confirmed COVID-19 [16] in the hospital, and there were no restrictions on SARS-CoV-2 PCR testing. Third, age, sex, and chronic diseases were homogeneously distributed among blood groups, and none of the participants had been vaccinated against COVID-19. This may have helped the authors rationally evaluate the relationship among blood groups, COVID-19 and disease severity. However, this study had some limitations. First, the results of the study, which was conducted at a single center and was retrospective in design, may not be generalizable. Second, the SARS-CoV-2 antigen was not tested in HCWs, so patients with asymptomatic COVID-19 were not excluded from the study. Finally, the relationship between hospitalization, the need for ICU admission and deaths due to COVID-19 and ABO/Rh blood groups could not be demonstrated because of the small number of hospitalized patients.

## Conclusion

Our findings suggest that blood groups are associated with the development of SARS-CoV-2 infection in HCWs. Although the existence of a relationship between blood groups and the development of SARS-CoV-2 infection has been determined, we believe that more comprehensive studies are needed to understand the causative pathophysiological mechanisms.

**Summary points**

- SARS-CoV-2 infection developed at a rate approximately 20% lower rate in blood group O (15.6 vs 23.5%; odds ratio: 0.808; 95% CI: 0.655–0.996; p = 0.045).
- The rate of patients hospitalized due to SARS-CoV-2 infection in blood group O was found to be lower than that of the nonhospitalized group (25 vs 29.5%).
- The prevalence of blood group A was higher in hospitalized patients than in outpatients, but it was not statistically significant (50 vs 43.8%; p = 0.519).
- Although the risk of SARS-CoV-2 infection was lower in those who had anti-A compared with those who did not, no statistically significant difference was found (48.6 vs 51.1%; p = 0.308).
